# Between Clicks and Care: Investigating Social Media Addiction and Work Engagement Among Nurses in Saudi Arabia

**DOI:** 10.3390/nursrep15030084

**Published:** 2025-02-28

**Authors:** Zahour Ismael Boukari, Naglaa Abdelaziz Mahmoud Elseesy, Ohood Felemban, Ruba Alharazi

**Affiliations:** 1 Jeddah Second Health Custer, King Fahad General Hospital, Jeddah 23325, Saudi Arabia; zboukari@stu.kau.edu.sa; 2 Public Health Nursing Department, Faculty of Nursing, King Abdulaziz University, Jeddah 21589, Saudi Arabia; naalsesei@kau.edu.sa (N.A.M.E.); ofelemban@kau.edu.sa (O.F.); 3 Faculty of Nursing, Alexandria University, Alexandria 21527, Egypt; 4 Medical Surgical Nursing Department, Faculty of Nursing, King Abdulaziz University, Jeddah 21589, Saudi Arabia

**Keywords:** social media, networking, addiction, work engagement, nurses, healthcare, relationship

## Abstract

**Background/Objectives**: The aim of this study was to investigate the relationship between social media networking addiction (SMNA) and work engagement (WE) among nurses at a government hospital in Jeddah, Saudi Arabia, as well as to assess the overall levels of SMNA and WE within this population. **Methods**: A quantitative, correlational, cross-sectional design was employed with an online survey instrument, involving 283 nurses from the government hospital. The data were analyzed using SPSS software version 26. **Results**: Nurses exhibited low levels of SMNA and average levels of WE. A negative relationship was identified between SMNA and WE. **Conclusions**: The findings indicate that SMNA influences various dimensions of WE differently. A significant negative relationship was observed between SMNA and the dimensions of vigor, absorption, and overall WE. However, SMNA did not significantly impact the dedication dimension, which emerged as the highest-scoring aspect among nursing professionals. Therefore, this study recommends raising awareness among nurses regarding the detrimental impact that excessive social media usage can have on their professional responsibilities and mental health, the implementation of clear social media usage guidelines, regular assessment of the trends in social media usage among nurses and the introduction of educational programs to raise awareness and promote responsible usage.

## 1. Introduction

In today’s society, being “online” has become the norm; mobile phones and online networking have rapidly become integral to daily life, shaping how people connect, communicate, and consume information [1,2]. The prevalence of social media has reached remarkable levels, with around 4.59 billion users globally dedicating an average of 151 min each day to these platforms [3]. In Saudi Arabia, more than 51% of individuals utilize social media for health-related activities, with WhatsApp and Twitter emerging as especially favored platforms [4], and the average daily usage time was 3 to 4 h [5]. Among social media users, 54.1% were men and 45.9% were women [6].

Social networking sites (SNSs) have become essential components of contemporary communication and social engagement, transforming the ways in which individuals connect and exchange information [7]. SNSs are “virtual communities where users can create individual public profiles, interact with real-life friends, and meet new people” [2]. Social media networking platforms such as Facebook, Twitter, WhatsApp, Instagram, and Snapchat enable users to develop profiles, establish connections, and participate in a range of social activities [8]. However, communication on social media is double-edged. While it provides numerous benefits, excessive use can have adverse health consequences and may lead to addiction [9]. The increased usage has raised concerns regarding SNS addiction, as certain users display indications of psychological dependence [10,11]. SNS addiction can adversely affect various aspects of life, including social engagements, educational pursuits, professional responsibilities, interpersonal relationships, and mental health, the detrimental effects of SNS usage encompass reduced participation in real-world social activities, lower academic performance, and difficulties in personal relationships [12]. For healthcare workers, social media platforms serve as valuable tools for acquiring and sharing knowledge, connecting with colleagues, and supporting professional growth [13]. Nevertheless, excessive use of social media during working hours can pose risks to patient care quality [14]. This overuse may reduce the time and energy devoted to patient-centered tasks, diverting focus toward socializing or recreational activities and ultimately diminishing work performance [15]. The Ministry of Health (MOH) in Saudi Arabia developed the Transformation Strategies as part of “Vision 2030” for the Kingdom of Saudi Arabia [16]. A primary goal of the MoH within this strategy is to improve healthcare by enhancing service quality and consistency, as well as strengthening the performance and accountability of healthcare organizations and professionals. This approach aims to ensure the delivery of care that is safe, effective, timely, equitable, and patient-centered [16].

Previous research has explored the utilization of social media by healthcare professionals in Saudi Arabia, including the study conducted by Alghamdi et al. (2022) [17]. However, it primarily addresses broad usage trends and their general effects on job satisfaction. The current study builds upon this foundation by investigating the relationship between Social Media Network Addiction (SMNA) and work engagement specifically among nurses, providing more detailed insights and thus making a significant contribution to the existing body of knowledge. Another study regarding this was conducted by Alfifi et al. (2019) on the levels and factors of work engagement among nurses in hospitals in Najran, Saudi Arabia. The study reported that 49% of nurses were averagely engaged, with personal attributes and organizational factors positively related to WE; however, it did not explore the relationship between social media networking addiction and work engagement [18].

Moreover, recent studies reveal the significant impacts that controlled use of social media, supportive work environment, and job crafting behavior may have in reinforcing nurses’ work engagement as key factors contributing to nursing shortage and poor-quality care [19,20,21,22]. Thus, this study is significant for the health sector as it offers recommendations for healthcare organizations aimed at promoting employee well-being and enhancing organizational effectiveness such as the development of policies, educational programs, and a supportive environment aimed at enhancing engagement and managing social media addiction.

Although social media addiction has been extensively studied across various countries, there has been a lack of research in Saudi Arabia examining the relationship between social media networking addiction (SMNA) and work engagement (WE) among nurses. Moreover, most studies conducted in other countries have focused on the broader effects of social media addiction on mental health or job satisfaction, with significantly fewer investigations exploring its impact on work-related outcomes such as engagement, which is essential in fields that require high levels of commitment, energy, and focus. For example, Zhang et al. (2023) have shown that social media addiction has negatively affected work engagement; they refer mostly to broader concepts, such as job satisfaction or emotional exhaustion [23]. In addition, Ariani et al. (2020) investigated the effects of social media addiction on the performance of Indonesian nurses, similar to the focus on engagement in this study, yet they did not specifically explore its impact on vigor or absorption subdimensions [24]. Their findings indicated that employees who were highly engaged with social media during working hours were likely to be less engaged with work and that excessive use led to distractions and negatively affected the overall focus and productivity of employees at work.

However, this study addresses a gap in the existing literature by investigating the relationship between SMNA and WE among nurses in a government hospital in Jeddah, Saudi Arabia, and assessing the overall levels of SMNA and WE within this population. The current study also adds to the body of knowledge as it specifically breaks down work engagement into its subdimensions, namely vigor, absorption, and dedication, and then assesses the relationship between the SMNA and each of them. In addition, it focused on nurses in Jeddah, introducing a new perspective on how different environments and cultural backgrounds may influence social media-related behaviors. The study particularly highlights the cultural context of Saudi Arabia, where social media usage patterns and work practices in healthcare settings may differ from those in Western countries [4]. This localized perspective enriches the global discourse on social media addiction in the workplace and provides relevant insights for similar contexts, such as other regions in the Middle East or areas with analogous healthcare systems. Furthermore, this study specifically targets nurses, offering valuable insights into the effects of social media addiction on healthcare professionals’ performance due to the unique challenges associated with their profession. Nursing is marked by substantial psychological pressure, emotional demands, and a high level of responsibility; consequently, staff may experience diminished professional engagement and increased susceptibility to social media distractions.

## 2. Materials and Methods

### 2.1. Study Design

This study employed a quantitative, correlational, cross-sectional research design to investigate the relationship between SMNA and WE among nurses at a governmental hospital in Jeddah, Saudi Arabia. This study design was chosen as it facilitates the observation and analysis of the relationships between variables, specifically SMNA and WE, without the need to manipulate or control the environment or the participants. The research utilized self-reported data gathered via an online survey, which aligns well with the objective of investigating existing patterns and relationships within the nursing population.

### 2.2. Study Setting and Participants

Data were collected using Google Drive surveys distributed to a random sample of 283 nurses, selected from a total population of 1062 nurses employed at the governmental Hospital in Jeddah, Saudi Arabia. The sample consisted of both male and female nurses from various departments at the governmental hospital. The sample size was calculated using the Raosoft Power Analysis online program, ensuring a 5% margin of error and a 95% confidence interval. Based on this calculation, the targeted sample was 283 participants. The researcher personally introduced herself to the head nurses of each hospital department and requested their assistance in distributing the questionnaire in order to facilitate the data collection process and gather as many responses as possible, especially since the head nurse in each department has direct contact with their nursing staff, has all their contact details and can easily reach them. In addition, the researcher distributed the questionnaires by herself in person by providing printed questionnaires, displaying a printed barcode on announcement boards, or sharing a link to the questionnaire via WhatsApp.

#### Inclusion and Exclusion Criteria

The inclusion criteria encompassed all adult nursing staff, both female and male, of various nationalities, working in different clinical areas including ambulatory services, inpatient units, and critical care at the governmental hospital. Exclusion criteria included students, such as intern nurses, who were excluded due to their limited clinical involvement and potential confounding factors affecting the study results. Additionally, nurses in managerial roles, including first-line managers, supervisors, and nursing directors, as well as those in education and quality departments, were excluded because their roles often require frequent mobile application use during work hours, which could influence the study outcomes. Other healthcare coworkers were also excluded, as the study specifically focused on nursing specialty.

### 2.3. Data Collection Method

Data were collected during the period from February 2024 to June 2024 through a self-administered questionnaire consisting of three main sections:

Part I: Nurses’ Sociodemographic Data

This section, developed by the researcher, included eight items to assess participants’ sociodemographic characteristics. These included age, gender, nationality, marital status, level of education, years of experience in the nursing field, unit assignment, and working shift.

Part II: Assessment of Social Networking Addiction

The Social Networking Addiction Scale, developed by [9] based on [25] component model, was used to assess SMNA. The scale comprises 21 items divided into six subscales: salience (4 items), mood modification (3 items), tolerance (3 items), withdrawal (4 items), conflict (3 items), and relapse (4 items). Responses were recorded using a 7-point Likert scale ranging from “strongly disagree” to “strongly agree”. Scores range from 21 to 147, with scores above 84 indicating addiction and scores below 84 signifying no addiction. Additionally, the questionnaire included a question to identify the most widely used social networking application among participants. This provided valuable insights into platform preferences and usage patterns.

Part III: Work Engagement Scale

Work engagement was assessed using the Utrecht Work Engagement Scale (UWES), developed by [26]. This scale consists of 17 items measured on a 7-point Likert scale, ranging from “never” (0) to “always” (6). The scale evaluates three dimensions of work engagement:

1. **Vigor (6 items):** Measures energy levels, mental concentration, effort, and persistence in facing challenges.
2. **Dedication (5 items):** Assesses a sense of significance, enthusiasm, inspiration, and pride in work.
3. **Absorption (6 items):** Evaluates full immersion in work, with a perception of time passing quickly and reduced distraction.

The overall score on the UWES ranges from 0 to 102. Mean scores for each subscale were calculated by dividing the sum of the subscale scores by the number of items. Five engagement categories were defined: very low (≤1.93), low (1.94–3.06), average (3.07–4.66), high (4.67–5.53), and very high (≥5.54) [5].

Moreover, the researcher utilizes both content and construct validity to ensure that the tool measures only the intended variables. The questionnaire was adopted from previously developed and validated scales; the Social Networking Addiction Scale (SNAS) which was developed by Shahnawaz and Rehman in 2020 [9], and the Utrecht Work Engagement Scale (UWES) developed by Schaufeli and Bakker in 2004 [26], thereby preserving their validity. In addition, a panel of five experts from the Nursing Faculty at King Abdulaziz University in Jeddah conducted the validity assessment. The specialists reviewed the items and determined the assessment methodology for each one. To ensure internal consistency, a Pearson simple correlation coefficient was calculated. The results indicate that each item is statistically significant to the total score of its respective axis, demonstrating that the tool has a high degree of internal consistency validity.

Furthermore, the reliability of the study tools was assessed using Cronbach’s alpha coefficient. Table 1 shows the reliability test results using Cronbach’s alpha. The coefficient for the Social Media Networking Addiction Scale (SNAS) was 0.95, and for the **Utrecht Work Engagement Scale** (UWES) **was** 0.95. Both values exceed the 0.7 threshold, indicating high reliability for the measurement tools. Due to non-normal distribution of scores in both scales and their domains, non-parametric tests were applied, with statistical significance set at *p* < 0.05. 

In addition, a pilot study was conducted in the actual study setting to establish Cronbach’s alpha coefficient and internal consistency validity for this study. Furthermore, to ensure that the scales were culturally and linguistically suitable for the Saudi Arabian context and for Saudi nurses who may have limited proficiency in English, they were translated into Arabic using established translation and back-translation procedures. This method ensured that the meaning of each item was maintained while making the scales accessible and relevant to Arabic-speaking participants. The translation and back-translation process were conducted between December 2023 and January 2024. The back-translation process was performed by professional bilingual translators who were fluent in both English and Arabic to ensure conceptual equivalence. Additionally, the translated scales underwent pretesting with a small group of Saudi nurses to confirm their clarity, relevance, and comprehensibility. Feedback from the pretesting phase was incorporated to refine the final versions of the scales, ensuring linguistic and contextual appropriateness. Furthermore, the validity and reliability of the Saudi versions of the scales were ensured by conducting an expert review, which involved five nursing experts to assess the content clarity and relevance of the scale. Additionally, internal consistency was evaluated using Cronbach’s alpha coefficient, which obtained score of 0.95 indicating high reliability.

### 2.4. Data Analysis

Data were analyzed using Statistical Package for the Social Sciences (SPSS) software version 26.0 (IBM Corp. Released 2019. IBM SPSS Statistics for Windows, Version 26.0. Armonk, NY, USA: IBM Corp.) [27]. The reliability of the study tools was assessed using Cronbach’s alpha coefficient. The normality of the data distribution was tested using the Kolmogorov–Smirnov test. Descriptive statistics were presented as numbers and percentages for categorical variables and as means and standard deviations for continuous variables. The relationship between SMNA and WE was assessed using the Mann–Whitney U test. Additionally, the relationships between SMNA, WE, and sociodemographic characteristics (e.g., age, gender, years of experience) were analyzed using the Chi-square test. The statistical methods utilized in this study were crucial for ensuring the reliability, validity and consistency of the study instruments [28].

### 2.5. Ethical Consideration

The researcher obtained permission from the Research Ethical Committee of the Nursing Faculty at King Abdulaziz University in Jeddah (registration no. 1M. 16, 24 July 2023). Additionally, the study received ethical approval from the Committee of the Research and Studies Department at the Directorate of Health Affairs in Jeddah, under the oversight of the Institutional Review Board (IRB) (IRB no. A0179, approved date 6 December 2023). Informed consent was obtained from participants. In this study, there was no real or possible risk associated with participation. The researcher treated all collected information with anonymity and confidentiality, ensuring privacy by using the data solely for this research and keeping it securely in their possession.

## 3. Results

### 3.1. Participant Characteristics

Slightly more than half of the participants (54.4%) were between 30 and 39 years of age, with females comprising 86.2% of the sample. Nearly two-thirds of the participants (64.7%) were Saudi nationals, and 70.7% were married. More than half of the participants (62.5%) held a bachelor’s degree. Approximately 34.3% of the participants had between 10 and 14 years of experience in the nursing field. The most common unit of assignment was ambulatory services (47.3%), and the predominant working shift was the morning shift (61.5%).

### 3.2. Assessment of Social Media Networking Addictions Among Nurses

Regarding the assessment of the SNAS questionnaire, Table 2 shows the highest rating relating to the silence domain was, “I go to social networking sites instantly after waking up in the morning” with a mean score of 3.57 ± 1.89, while it was less in the statement, “While I work, my mind remains on social networking sites,” with a mean score of 2.33 ± 1.57. The top-rated statement for the mood modification domain was, “Social networking helps me lift my mood” (3.87 ± 1.76), while for the tolerance domain, “When compared, I spend more time on social networking sites now than I did in the past” (3.54 ± 1.76) was the top-rated statement. For the withdrawal domain, the statement with the highest rating was, “I feel sad when I am unable to log into social networking sites” (2.82 ± 1.69), while “I try to hide the time I spend on social networking.” (2.59 ± 1.55) showed the highest rating for the conflict domain. Finally, the statement with the highest rating for the relapse domain was, “I have tried to stop using social networking sites but failed.” (2.89 ± 1.65).

Table 3 presents the descriptive statistics for the SNAS domains. The highest mean score was observed in the mood modification domain, with a mean percentage of 53.3% and a score of 11.2 ± 4.84. This was followed by the tolerance domain (9.93 ± 4.66), then the salience domain with a mean score of 12.2 ± 5.94. Subsequently, the relapse domain had a mean score of 11.2 ± 5.95, while the withdrawal domain recorded a mean score of 10.6 ± 5.97. Finally, the lowest mean score was noted in the conflict domain at 7.10 ± 4.11, with a mean percentage of 33.8%.

Table 4 presents the overall mean percentage score of SNAS, which was 42.3%, with a mean score of 62.2 ± 25.6. Based on these results, 17.7% of the nurses were classified as having SMNA, while 82.3% of the nurses were classified as not having SMNA.

**Figure 1:**
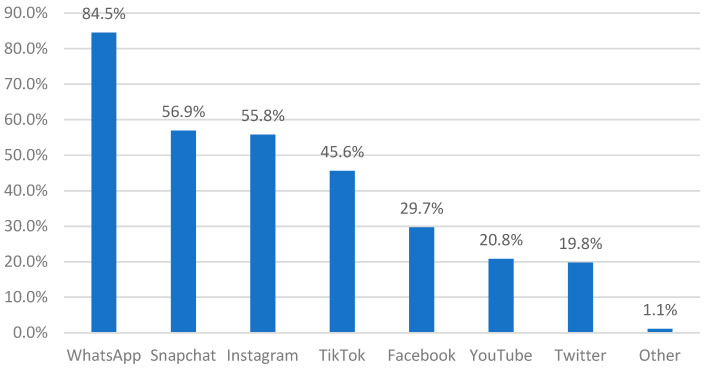
Most common social media platform usage.

Figure 1 illustrates the most commonly used social media platforms based on the responses from nurses, providing a visual representation of preferences and trends in social media usage among the participants. The most commonly used platform by nurses was WhatsApp (84.5%), followed by Snapchat (56.9%) and Instagram (55.8%). In contrast, YouTube (20.8%) and Twitter (19.8%) were the least used social media platforms.

### 3.3. Assessment of Nurses’ Work Engagement

Table 5 shows that the highest-rated item within the vigor domain was, “At my work, I always persevere, even when things do not go well” (4.15 ± 1.62), whereas the lowest-rated item was “I can continue working for very long periods at a time” (3.25 ± 1.84). For the dedication domain, the highest-rated statement was “I am proud of the work that I do” (4.72 ± 1.61), while the lowest-rated was “I am enthusiastic about my job” (4.36 ± 1.57). Finally, within the absorption domain, the top-rated statement was “Time flies when I’m working” (4.17 ± 1.60), although the lowest-rated item was “I feel happy when I am working intensely” (3.49 ± 1.85).

Table 6 presents the descriptive statistics for the domains of the UWES. The findings reveal that the highest mean percentage score for WE was observed in the dedication domain 75.7% (4.54 ± 1.46), followed by the average level of absorption domain which was 64% (3.84 ± 1.43) and the vigor domain which was 61.5% (3.69 ± 1.36). Furthermore, the overall mean percentage score for the UWES was an average of 66.5% (3.99 ± 1.22).

Table 7 the analysis found that social media addiction was inversely correlated with the vigor domain (Z = −1.983; *p* = 0.047). Similarly, a significant negative correlation was observed with the absorption domain (Z = −2.407; *p* = 0.016). Furthermore, there was a statistically significant inverse correlation with the overall WE score (UWES), as indicated by Z = −2.277; *p* = 0.023. Conversely, no significant association was found between SMNA and the dedication domain (*p* = 0.752).

Table 8 the analysis of the relationship between SMNA and WE, based on the socio-demographic characteristics of the nurses, revealed that male gender (X^2^ = 16.966; *p* < 0.001) and Saudi nationality (X^2^ = 7.987; *p* = 0.005) were significantly associated with higher levels of SMNA. In terms of WE, the only socio-demographic factor showing a significant relationship was the unit of assignment, indicating that nurses working in inpatient units were more likely to exhibit low to average levels of WE (X^2^ = 6.219; *p* = 0.045). However, no significant associations were found between SMNA and WE with the other socio-demographic factors (all *p* > 0.05).

## 4. Discussion

The sociodemographic analysis revealed that the majority of nurses were aged 30–39, reflecting an early to mid-career workforce. This observation aligns with the findings of Lu et al. [29], as the average age of nurses typically falls within this bracket. Furthermore, over two-thirds of the participants were female, consistent with gender trends commonly observed in the nursing profession [30]. A substantial proportion of the sample (approximately two-thirds) consisted of Saudi nationals, aligning with recent initiatives to enforce “Saudization” policies which have aimed at increasing the representation of Saudi nurses within the healthcare sector [31]. Additionally, more than two-thirds of nurses were married, balancing family and professional roles. In terms of educational qualifications, the majority of the nurses (nearly two-thirds) held a bachelor’s degree, reflecting a prevalent trend in advanced healthcare systems where undergraduate education serves as the foundational requirement for professional practice [32]. A significant portion of the workforce (about one-third) had between 10 and 14 years of experience, indicating a significant presence of experienced professionals within the study sample. The ambulatory services unit, which accounted for over half of the participants, emerged as the most common area of employment, likely due to its flexible scheduling options [33]. Furthermore, nearly two-thirds of the nurses worked morning shifts, which may have facilitated their ability to participate in the study due to better energy levels and availability after their duties. These results align with the study conducted by Friese et al. (2016), in which a majority (79.2%) of participants were nurses working in an ambulatory unit [34].

Moreover, the study employed six variables to assess social media addiction: salience, mood modification, tolerance, withdrawal, conflict, and relapse. Each variable represents a distinct aspect of how nurses interact with and depend on social media. The highest mean scores were associated with mood modification, particularly regarding the statements “I go to social networking sites whenever I am upset” and “Social networking helps me lift my mood”. These findings are consistent with previous studies [35], which indicate that social media is frequently used for emotional regulation. The dimensions of tolerance and mood modification also scored notably high, suggesting that while nurses may not exhibit severe addiction, their engagement with social media serves as a coping strategy for stress [36]. In contrast, the lowest-rated statement was related to the conflict domain which involves interpersonal issues arising from social media use. The statement “I need to lie to my parents and others when they ask about my social networking usage” had a particularly low mean score, suggesting that social media use among nurses does not typically create interpersonal conflicts that require deception. This may be attributed to the fact that most participants are independent adults, with many likely being married, which reduces the need to hide their social media use. It indicates that while nurses may use social media for emotional regulation, it does not significantly interfere with their relationships, contributing to the overall low level of addiction.

Based on the results, the study found that less than a quarter of participants were classified as addicted, while more than three-quarters were classified as non-addicted. This finding indicates a low level of SMNA among nurses. The study’s findings suggest that while most participants are not addicted to social media, a smaller but significant group does require targeted intervention. In addition, the demanding nature of nursing work, often characterized by long or irregular shifts, limits the time available for prolonged social media use. This may contribute to the overall low level of SMNA observed among nurses. Furthermore, the high level of attention and responsibility required in nursing further restricts opportunities to engage in extensive social media use during working hours. This finding aligns with the existing literature, which highlights varying levels of social media use among healthcare professionals. Similarly, a study by Hoşgör et al. [20] reported that participating nurses exhibited low levels of social media addiction. In contrast, Luo et al. [37] found that social media addiction scores were relatively high among Chinese healthcare workers. Likewise, Sulasula [38] reported that 70% of respondents among Philippine government personnel showed moderate to high levels of SMNA. Another study [39] found that **nomophobia**, defined as the fear of being without a smartphone, was generally observed at a moderate level among nurses.

The survey results indicate the most used social media platforms among the nurses participating. WhatsApp emerged as the most frequently used platform, with usage reported by more than three-quarters of the respondents. This was followed by Snapchat and Instagram, which were used by over half of the participants. In contrast, YouTube and Twitter were the least utilized platforms, with usage rates reported by less than a quarter of the respondents. The preference for WhatsApp may be attributed to its ease of use and widespread adoption for quick, efficient communication, making it a convenient tool for both personal and professional interactions. Snapchat and Instagram, being visually driven platforms, might appeal to nurses looking for entertainment-oriented content. The lower usage of YouTube and Twitter could be due to the time-consuming nature of video content and the text-heavy format of Twitter, which may not align well with the preferences or time constraints of the participants. These results are aligned with El Kheir’s [4] study, which found that the population of Saudi Arabia has demonstrated a strong preference for utilizing WhatsApp and Twitter as platforms for acquiring and sharing information.

The study assessed WE among nurses using three key dimensions: vigor, dedication, and absorption. The results indicated relatively similar scores across these dimensions. Notably, the dedication dimension achieved the highest score, with nurses expressing a sense of pride in their work, which aligns with the perception of nursing as an honorable profession dedicated to providing meaningful and purposeful work [40]. Conversely, in the vigor domain, the item “I can continue working for very long periods at a time” received the lowest rating, suggesting that while nurses exhibit dedication, sustaining energy and enthusiasm over prolonged periods may frequently experience burnout or fatigue as a result of extended shifts and substantial patient loads [41]. The study revealed that the dedication domain had the highest mean score for work engagement. Dedication is defined as the level of commitment and enthusiasm nurses feel toward their work, suggesting that many nurses find meaning and purpose in their roles [42]. The absorption domain, reflecting the extent to which nurses are deeply engaged and immersed in their tasks [43] was reported at an average level, indicating moderate focus and involvement in work activities. Similarly, the vigor domain, which represents the energy and enthusiasm nurses bring to their roles [43] also scored at an average level. Participants generally exhibited moderate energy and mental resilience, though they struggled to sustain high energy levels over time. These findings align with the existing literature, which frequently reports higher dedication scores compared to vigor among healthcare professionals. For instance, Ghazawy et al. [44] found that in Egypt, dedication scored higher than both vigor and absorption. Similarly, Alharbi and Alrwaitey [45] identified dedication as the highest dimension of WE in Saudi Arabia, followed by absorption and vigor.

The analysis further showed that nearly half of the nurses reported an average WE level. This may reflect a work environment characterized by moderate support, recognition, and opportunities for professional growth. Organizational policies and workloads likely provide stability but fail to strongly incentivize higher engagement levels. The average levels of engagement identified in this study are consistent with those reported in comparable studies, such as those by Alharbi and Alrwaitey [45], García-Iglesias et al. [46], Xue et al. [47], and Zhou et al. [48], which indicated that nurses across various global contexts often report moderate levels of work engagement. These similarities suggest that, despite regional variations, the factors influencing nurses’ engagement such as job demands, organizational support, and workload tend to be universal. Conversely, Diab and El Nagar [49] reported that two-thirds of their participants demonstrated low levels of WE, highlighting a negative association between job stress and engagement. Therefore, WE among nurses may tend to decline when they face challenges such as increased workload, inadequate compensation, insufficient support, or difficulty adapting to environmental changes.

The current study revealed that one-third of the participants fell into the “high to very high” engagement category, while nearly one-quarter reported “low to very low” engagement. These variations in WE level among the studied nurses could be attributed to differences in intrinsic motivation, career aspirations, personal challenges, burnout, workplace stressors, or satisfaction with the work environment. Supporting these findings, Liebenberg et al. [50] conducted a study to examine the daily variation in nurses’ WE over a 7-day period. Liebenberg et al. [50] identified positive relationships between the daily satisfaction of competence needs and variations in WE among nurses, noting that general emotional load affects daily competence variability. Additionally, Nguyen et al. [51] discovered that changes in nursing work were found to cause a higher workload and an increase in administrative stressors, which contributed to greater cynicism toward organizational change among nurses. Cynicism about organizational change was found to have a direct negative impact on nurses’ engagement, which subsequently led to lower job satisfaction. A study conducted by Wei et al. [52] reported that nurses’ WE levels were significantly influenced by job satisfaction, perceived quality of care, and their intention to leave. The importance of purpose and value in professional roles has been emphasized in other research. Nurdiyansyah et al. [53] highlighted that employees who perceive their work as meaningful are more motivated, respected, and engaged, enhancing their satisfaction and commitment. Hara et al. [54] demonstrated a positive correlation between WE and the perceived attractiveness of working in nursing, where occupational commitment, mediated by WE, was influenced by both the attractiveness of the work and autonomous clinical judgment. Furthermore, Sheehan et al. [6] observed that higher levels of WE were associated with positive perceptions of high-involvement work practices (HIWPs). These perceptions were also positively linked to job crafting, which mediated the indirect effect of HIWPs on WE. Moreover, Li et al. [55] found that nurses with formal employment exhibited higher engagement levels compared to those without, benefiting from greater stability, wage security, and career prospects. In contrast, non-staff nurses faced uncertain career paths, potentially reducing their engagement.

A further investigation was conducted to assess the relationship between SMNA and WE among nurses. The analysis revealed a negative correlation between SMNA and the overall WE. These findings align with the existing literature, which highlights a significant negative correlation between SMNA and WE among nurses. For instance, a study by Hoşgör et al. [20] demonstrates that SMNA negatively impacts WE. Among government employees, the results are consistent with the findings of Sulasula [38], who identified a negative relationship between SMNA and WE.

The current study analyzes the relationship between social media addiction and the WE subscale used in the study, including **vigor, absorption,** and **dedication**. The results indicate that social media addiction affects different dimensions of WE in varied ways. The analysis found that social media addiction was inversely correlated with the vigor domain. Similarly, a significant negative correlation was observed with the Absorption domain. Conversely, no significant association was found between social media addiction and the dedication domain. These findings suggest that social media addiction may contribute to decreased levels of energy and immersion at work, as reflected in the vigor and absorption dimensions of work engagement. Social media use could delay immediate focus and provide mental stimulation, potentially causing individuals with addictive tendencies to feel less engaged or energized when attempting to concentrate on work tasks due to frequent distractions. Ibrahim et al. [42] and Pucciarelli et al. [56] support this explanation, noting that social media addiction increases the cognitive demands placed on employees, leading to recurrent distractions that diminish engagement. According to Ibrahim et al. [42], the addictive features of social media such as social comparison and constant notifications can disrupt employees’ focus, ultimately resulting in lower engagement. Additionally, Javed et al. [14] state that social media networking leads to task distraction, negatively affecting nurses’ performance and further compounding this issue.

The study investigates the relationship between SMNA and WE across various socio-demographic characteristics of nurses, including age, gender, nationality, marital status, educational level, years of work experience, unit of assignment, and working shift. The findings indicate that gender and nationality were significantly associated with SMNA. Furthermore, the results reveal that the unit of assignment was significantly related to WE. The findings reveal a significant relationship between gender and SMNA. Specifically, findings revealed that male nurses reported higher levels of SMNA compared to their female counterparts, a finding that contradicts previous research, such as those by Hussain et al. [57], Su et al. [58], Choudhury and Ali [59], which indicated a greater susceptibility to social media addiction among women. These discrepancies may stem from cultural influences or varying patterns of social media usage. For instance, males in Saudi Arabia may be more inclined to engage with technology and sports-related content, while females might utilize social media primarily for social interaction or entertainment purposes [60]. The findings indicate a significant relationship between nationality and SMNA among nurses, with Saudi nurses exhibiting higher addiction levels compared to their non-Saudi counterparts. This observation corroborates previous studies highlighting the impact of cultural and societal influences on social media behaviors [61]. In Saudi Arabia, social media is extensively utilized and intricately woven into daily life, individuals in Saudi Arabia dedicate an average of four hours each day to social media platforms [62]. This elevated usage rate likely reflects both the cultural importance of social media and the interpersonal dynamics within the workplace, where nurses may allocate considerable time to connect with family and social networks. The findings of the current research indicate a significant relationship between the unit of assignment and WE level among nurses. Specifically, nurses assigned to inpatient units are more likely to exhibit low to average levels of engagement compared to those working in critical care or ambulatory services; however, no statistically significant relationship was found between these unit types and engagement levels. This disparity may be attributed to the distinct work environments, task structures, workloads, and demands associated with each unit type. The higher engagement levels observed among nurses in ambulatory services may be attributed to the regular and predictable work hours typical of these settings, which foster greater engagement. Similarly, the engagement levels of critical care nurses may be linked to their vital role in saving lives and improving patient outcomes, thereby reinforcing their sense of purpose. In contrast, inpatient nurses often manage higher patient ratios with diverse and complex needs, which may contribute to these differences in engagement levels. These findings are consistent with the study conducted by Chiang and Chang [63] who reported that nurses in medical and surgical departments exhibited significantly lower levels of vigor and dedication compared to their counterparts in ambulatory and outpatient clinics. Similarly, García-Iglesias et al. [46] observed emergency nurses experienced heightened levels of psychological risk and discomfort, whereas primary care nurses exhibited higher engagement across all three dimensions of work engagement. The analysis of socio-demographic factors in the current research did not identify a relationship between WE and other variables such as gender, education, marital status, work experience, or working shifts. However, previous studies, such as those by Bakertzis and Myloni [64] and Mori et al. [65], have identified a significant relationship between WE and age, suggesting that older healthcare providers tend to exhibit higher levels of WE compared to their younger counterparts.

### 4.1. Limitations

This study encountered limitations related to the data collection process as the study was carried out in a single setting in the geographical area of Jeddah in Saudi Arabia, which hindered the generalizability of the results beyond the specific context of the study. It is crucial for the subsequent research to be conducted across multiple sites and diverse settings, including other locations, and adopting a mixed-methods approach for comprehensive data and generalization of results. In addition, data for this study were collected using a self-administered questionnaire survey of nurses. Such surveys are prone to biases, including social desirability, self-exaggeration, and underreporting due to embarrassment. To mitigate these biases, considerable efforts were made to anonymize responses and ensure participants’ privacy. However, it remains challenging to completely eliminate all potential sources of bias, particularly given the sensitivity of the study variables.

### 4.2. Recommendations

Based on the study findings, the recommendations from this study are to raise awareness among nurses regarding the detrimental impact that excessive social media usage can have on their professional responsibilities and mental health. In addition, continuous assessment of the trends in social media usage among nurses is necessary to gauge the extent of addiction, enabling the implementation of appropriate interventions to mitigate any further escalation. Another measure that could promote WE is developing clear guidelines for social media usage during work hours.

Moreover, further study is recommended to explore these relationships among the involved variables and to develop interventions that promote healthier social media habits within the nursing field. In addition, future studies need to investigate the relationship between social media networking addiction (SMNA) and work engagement (WE) across various healthcare institutions in KSA, including both public and private hospitals, to provide broader insights and reinforce the findings presented.

## 5. Conclusions

This study investigated the relationship between social media networking addiction (SMNA) and work engagement (WE) among nurses at a government hospital in Jeddah, Saudi Arabia using an online questionnaire. In this study, a 7-point Likert scale was used for both the Social Networking Addiction Scale and the Utrecht Work Engagement Scale which was a well-established method to provide more depth and nuance in the responses, thus offering a clearer understanding of participants’ perspectives. These scales adopted from a previous study have been validated in prior research. The statistical analysis was based on descriptive statistics and tests such as the use of Mann–Whitney U test, Chi-square test, Kolmogorov–Smirnov test, Pearson’s correlation and Cronbach’s alpha coefficient which was 0.95, indicating high reliability for the measurement tools.

The findings of the study revealed that nurses exhibited low levels of SMNA and average levels of WE. A significant negative relationship was found between SMNA and WE particularly impacting the vigor and absorption dimensions, while dedication remained unaffected and was the highest-scoring aspect. This indicates that the majority of nurses do not exhibit an addiction to social media. Moreover, the study identified socio-demographic variations, revealing that male nurses and Saudi nationals reported higher levels of Social Media Network Addiction (SMNA). Additionally, it was found that the specific unit of assignment influenced levels of work engagement (WE), with nurses in ambulatory services and critical care exhibiting greater engagement compared to those in inpatient units. Thus, nurses could greatly benefit from specific interventions aimed at preventing excessive usage. This necessitates the implementation of clear policies governing social media use, regular assessments, and initiatives promoting digital detoxification to ensure a healthy balance between professional duties and time allocated to social media platforms, thereby suggesting implications for workplace policies aimed at enhancing well-being, productivity, and job satisfaction among nursing professionals. In conclusion, the findings indicate that the research objectives were achieved, yielding an in-depth understanding of the relationship between social media networking addiction (SMNA) and work engagement (WE) among nurses in Jeddah, KSA.

## Figures and Tables

**Table 1:** Cronbach’s alpha coefficient.

N	Domain	No. of	Cronbach’s Alpha Coefficient
1	The Social Media Networking Addiction scale	21	0.95
2	The UWES	17	0.95

**Table 2:** Social media networking addictions self-assessment tools (n = 283).

Silence	Mean ± SD	Mean%
While I work, my mind remains on social networking sites	2.33 ± 1.57	33.29%
2. I go to social networking sites instantly after waking up in the morning.	3.57 ± 1.89	51.0%
3. I check updates on social networking sites while working.	3.17 ± 1.77	45.29%
4. I check my social networking account before starting any task or activity.	3.08 ± 1.82	44.0%
**Mood modification**		
5. I go to social networking sites whenever I am upset.	3.78 ± 1.79	54.0%
6. Social networking helps me lift my mood.	3.87 ± 1.76	55.29%
7. I feel relaxed whenever I am on social networking sites.	3.54 ± 1.75	50.57%
**Tolerance**		
8. These days, I spend more and more time on social networking sites.	3.52 ± 1.74	50.29%
9. When compared, I spend more time on social networking sites now than I did in the past.	3.54 ± 1.76	50.57%
10. I need to be on social networking sites for longer time than before to be satisfied	2.88 ± 1.69	41.14%
**Withdrawal**		
11. I feel sad when I am unable to log into social networking sites	2.82 ± 1.69	40.29%
12. I become irritable whenever I cannot log in to social networking sites.	2.58 ± 1.58	36.86%
13. I feel frustrated when I cannot use social networking sites.	2.66 ± 1.63	38.0%
14. I become restless when I do not get time for social networking.	2.53 ± 1.58	36.14%
**Conflict**		
15. I try to hide the time I spend on social networking.	2.59 ± 1.55	37.0%
16. I need to lie to my parents and others when they ask about my social networking usage.	2.12 ± 1.43	30.29%
17. I ignore my sleep because I have/want to be on social networking sites.	2.39 ± 1.67	34.14%
**Relapse**		
18. I have failed to cut down the time I spend on social networking sites.	2.77 ± 1.61	39.57%
19. I have tried to stop using social networking sites but failed.	2.89 ± 1.65	41.29%
20. I am unable to cut down on the time I spend on social networking sites.	2.78 ± 1.58	39.71%
21. My repeated attempts to reduce the time I spend on social networking sites have failed.	2.80 ± 1.69	40.0%

**Table 3:** The domains of social media networking addictions (n = 283).

SNAS Domain	Mean ± SD	Mean (%)	Perceived Level
Silence score	12.2 ± 5.94	43.6%	Low
Mood modification score	11.2 ± 4.84	53.3%	Average
Tolerance score	9.93 ± 4.66	47.3%	Average
Withdrawal score	10.6 ± 5.97	37.9%	Low
Conflict score	7.10 ± 4.11	33.8%	Low
Relapse score	11.2 ± 5.95	40.0%	Low
**SNAS total score**	**62.2 ± 25.6**	**42.3%**	**Low**

**Table 4:** Level of social media networking addictions (n = 283).

Level of Addiction	Score Range	N (%)	Perceived Level
Addiction	≥84	50 (17.7%)	Low
Non-addiction	<84	233 (82.3%)	High

**Table 5:** Descriptive statistics of work engagement scale (n = 283).

Vigor	Mean ± SD	Mean%
At my work, I feel bursting with energy	3.65 ± 1.69	60.83%
2. At my job, I feel strong and vigorous	3.76 ± 1.58	62.67%
3. When I get up in the morning, I feel like going to work	3.55 ± 1.62	59.17%
4. I can continue working for very long periods at a time	3.25 ± 1.84	54.17%
5. At my job, I am very resilient, mentally	3.81 ± 1.65	63.50%
6. At my work, I always persevere, even when things do not go well	4.15 ± 1.62	69.17%
**Dedication**		
7. I find the work that I do full of meaning and purpose.	4.53 ± 1.59	75.50%
8. I am enthusiastic about my job	4.36 ± 1.57	72.67%
9. My job inspires me	4.51 ± 1.63	75.17%
10. I am proud of the work that I do	4.72 ± 1.61	78.67%
11. To me, my job is challenging	4.60 ± 1.58	76.67%
**Absorption**		
12. Time flies when I’m working	4.17 ± 1.60	69.50%
13. When I am working, I forget everything else around me	3.82 ± 1.75	63.67%
14. I feel happy when I am working intensely	3.49 ± 1.85	58.17%
15. I am immersed in my work	4.04 ± 1.64	67.33%
16. I get carried away when I’m working	3.88 ± 1.68	64.67%
17. It is difficult to detach myself from my job	3.67 ± 1.85	61.17%

**Table 6:** Descriptive statistics of **Utrecht Work Engagement Scale** domain (n = 283).

UWES Domain	Mean ± SD	Mean (%)	Perceived Level
Vigor score	3.69 ± 1.36	61.5%	**Average**
Dedication score	4.54 ± 1.46	75.7%	**Average**
Absorption score	3.84 ± 1.43	64.0%	**Average**
**UWES total score**	**3.99 ± 1.22**	**66.5%**	**Average**
**UWES domain levels**	**Score range**	**N (%)**	

**Table 7:** Relationship between social media networking addiction and work engagement.

UWES Domain	Social Media Networking Addiction		Z-Test	*p*-Value ^§^
	Addiction Mean ± SD	Non-Addiction Mean ± SD		
Vigor score	3.23 ± 1.58	3.79 ± 1.28	−1.983	0.047 **
Dedication score	4.66 ± 1.29	4.52 ± 1.49	0.315	0.752
Absorption score	3.28 ± 1.65	3.96 ± 1.35	−2.407	0.016 **
UWES total score	3.49 ± 1.49	4.10 ± 1.13	−2.277	0.023 **

^§^ *p*-value has been calculated using Mann Whitney Z-test. ** Significant at *p* < 0.05 level.

**Table 8:** Relationship between social media networking addiction and work engagement among the nurses’ socio-demographic characteristics.

Factor	Social Media Addiction		Work Engagement	
	Addiction N (%) (n = 50)	Non Addiction N (%) (n = 233)	Low to Average N (%) (n = 191)	High/Very High N (%) (n = 92)
Age group				
<40 years	37 (74.0%)	156 (67.0%)	136 (71.2%)	57 (62.0%)
≥40 years	13 (26.0%)	77 (33.0%)	55 (28.8%)	35 (38.0%)
***X*^2^ ; *p-value*** ^§^	***0.943*; *0.332***		***2.448*; *0.118***	
Gender				
Male	16 (32.0%)	23 (9.9%)	24 (12.6%)	15 (16.3%)
Female	34 (68.0%)	210 (90.1%)	167 (87.4%)	77 (83.7%)
***X*^2^ ; *p-value*** ^§^	*16.966*; *0.001* **		***0.731*; *0.393***	
Nationality				
Saudi	41 (82.0%)	142 (60.9%)	121 (63.4%)	62 (67.4%)
Non-Saudi	9 (18.0%)	91 (39.1%)	70 (36.6%)	30 (32.6%)
***X*^2^; *p-value*** ^§^	*7.987*; *0.005* **		***0.444*; *0.505***	
Marital status				
Unmarried	18 (36.0%)	65 (27.9%)	54 (28.3%)	29 (31.5%)
Married	32 (64.0%)	168 (72.1%)	137 (71.7%)	63 (68.5%)
***X*^2^; *p-value*** ^§^	***1.304*; *0.253***		***0.316*; *0.574***	
Educational Level				
Diploma	11 (22.0%)	69 (29.6%)	49 (25.7%)	31 (33.7%)
Bachelor or higher	39 (78.0%)	164 (70.4%)	142 (74.3%)	61 (66.3%)
***X*^2^; *p-value*** ^§^	***1.177*; *0.278***		***1.980*; *0.159***	
Years of Working Experience				
<10 years	16 (32.0%)	71 (30.5%)	62 (32.5%)	25 (27.2%)
≥10 years	34 (68.0%)	162 (69.5%)	129 (67.5%)	67 (72.8%)
***X*^2^; *p-value*** ^§^	***0.045*; *0.832***		***0.815*; *0.367***	
Unit of Assignment				
Critical service	15 (30.0%)	58 (24.9%)	46 (24.1%)	27 (29.3%)
Inpatient units	10 (20.0%)	66 (28.3%)	60 (31.4%)	16 (17.4%)
Ambulatory service	25 (50.0%)	109 (46.8%)	85 (44.5%)	49 (53.3%)
***X*^2^; *p-value*** ^§^	***1.569*; *0.456***		*6.219*; *0.045* **	
Working Shift				
Morning	37 (74.0%)	137 (58.8%)	115 (60.2%)	59 (64.1%)
Evening/Night	2 (04.0%)	16 (6.9%)	12 (06.3%)	6 (06.5%)
Shifting	11 (22.0%)	80 (34.3%)	64 (33.5%)	27 (29.3%)
***X*^2^; *p-value*** ^§^	***4.027*; *0.134***		***0.495*; *0.781***	

^§^ *p*-value has been calculated using Chi-square test. ** Significang at *p* < 0.05 level.

## Data Availability

The data utilized in this study are available upon request from the corresponding author. However, they are not publicly accessible to ensure privacy.

## Public Involvement Statement

No public involvement in any aspect of this research.

## Guidelines and Standards Statement

This manuscript was drafted in accordance with the STROBE Statement for cross-sectional research.

## Abbreviations

The following abbreviations are used in this manuscript:

SMNA	Social Media Networking Addiction
SNAS	Social Media Networking Addiction Scale
WE	Work Engagement
UWES	Utrecht Work Engagement Scale
